# Prophylactic Retrorectus Mesh Versus Small-Stitch Closure After Emergency Midline Laparotomy: 2-Year Results of a Randomized Controlled Trial

**DOI:** 10.3389/jaws.2025.15500

**Published:** 2025-11-27

**Authors:** E. Mäkäräinen, M. Tolonen, V. Sallinen, P. Mentula, A. Leppäniemi, M. Ahonen, J. Saarnio, T. Pinta, H. Lampela, H. Malmi, E. Lietzen, M. Nikki, P. Ohtonen, F. Muysoms, T. Rautio

**Affiliations:** 1 Oulu University Hospital, Medical Research Center Oulu, Oulu, Finland; 2 HUS Helsinki University Hospital, Abdominal Center, Emergency Surgery, Helsinki, Finland; 3 Seinäjoki Central Hospital, Seinäjoki, Finland; 4 Department of Gastroenterological Surgery, University of Helsinki and Helsinki University Hospital, Helsinki, Finland; 5 Turku University Hospital, Turku, Finland; 6 Hospital AZ Maria Middelares, Ghent, Belgium

**Keywords:** incisional hernia, emergency laparotomy, midline laparotomy, incisional hernia prevention, randomized controlled trial

## Abstract

**Introduction:**

Incisional hernias (IH) are common complications following emergency midline laparotomies. Mesh reinforcement has shown efficacy in preventing incisional hernias in elective surgeries, but evidence remains limited for emergency midline incisions. This study aimed to evaluate the safety and effectiveness of retrorectus placement of a self-gripping polyester mesh in preventing incisional hernia after emergency midline laparotomy, as measured by the incidence of IH, postoperative complications, quality of life, and health economic outcomes.

**Methods:**

In this multicenter randomized controlled trial, adult patients undergoing emergency midline laparotomy were randomized to receive either prophylactic retrorectus mesh or standard 4:1 small-stitch fascial closure using a slowly absorbable monofilament suture. The primary outcome was the radiological/clinical IH rate within 2 years after the surgery. Secondary outcomes were complications, reoperations, quality of life, and health-economic measures. Blinding was maintained for patients, outcome assessors, and radiologists. Due to difficulties in recruitment, the study was prematurely terminated prior to reaching the aimed number of patients.

**Results:**

Out of 925 patients screened, 109 were randomized, and 72 received the allocated intervention. At 2-year’s follow-up, one (4%) asymptomatic incisional hernia was detected in the control group compared to none in mesh group. In the mesh group, three (9%) patients experienced mesh-related complications: one (5%) retrorectus hematoma, one (5%) internal hernia and one (5%) postoperative seroma. One (5%) additional patient in the mesh group developed a fistula requiring mesh removal. No significant differences were found in early postoperative complications or quality of life between groups.

**Conclusion:**

Retrorectus mesh reinforcement did not reduce the incidence of incisional hernia compared to standard small-stitch closure in this trial. However, mesh-related complications were observed. Due to recruitment challenges and limited sample size, definitive conclusions cannot be drawn.

**Clinical Trial Registration:**

https://clinicaltrials.gov/study/NCT04311788?term=preemer&rank=1, NCT04311788.

## Introduction

Emergency midline laparotomy is a risk factor for incisional hernia (IH), with up to 33% IH rate [1–4]. However, the guidelines do not provide any recommendations on midline closure after emergency laparotomy [5]. In elective setting, the recommended technique for midline fascial closure is the small stitch method, using a slowly absorbable monofilament suture with a suture-to-wound length ratio of at least 4:1 [5]. The same method can also be applied to close emergency midline laparotomy incisions to help prevent IH and fascial dehiscence [4, 6–8]. Additionally, prophylactic mesh augmentation in midline laparotomies has been both effective and safe for IH prevention after elective midline laparotomy [5, 9].

IH prevention after emergency midline laparotomy has been rarely studied. Ulutas et al. have published results of randomized controlled trial (RCT) using onlay mesh to prevent IH [6]. In the study, the preventive mesh decreased the IH rate significantly when compared to suture closure with small stitch technique (4% vs. 27%), without predisposing patients to increased risk of severe complications [6]. In another RCT, retrorectus mesh decreased the IH rate compared to small stitch closure (6% vs. 21%) [8].

The Preemer trial was designed as a multicenter RCT comparing retrorectus mesh-augmented closure with the conventional small-stitch 4:1 continuous fascial closure using a slowly absorbable monofilament suture for the prevention of incisional hernia after emergency midline laparotomy. In both groups, the fascia was closed in a continuous 4:1 manner using a slowly absorbable monofilament suture. The hypothesis was that the mesh prevents IHs compared to controls without increasing the risk of complications.

## Methods

### Trial Design

The PREEMER study was a multicenter, parallel-group, patient- and assessor-blinded, randomized controlled superiority trial conducted at Oulu, Helsinki, and Turku University Hospitals, as well as at the Central Hospital of Seinäjoki in Finland. The study aimed to determine whether prophylactic mesh reinforcement in emergency midline laparotomy closure is superior to standard primary closure in preventing incisional hernia without increasing postoperative complications.

The study protocol was published previously [10], and the trial was registered at ClinicalTrials.gov before enrollment started (NCT04311788). Eligible patients were recruited between 22 April 2020, and 10 October 2022. After receiving both oral and written information and providing written informed consent, patients were enrolled in the trial.

Participants were followed up at 30 days postoperatively, either by phone interview or at an outpatient clinic, to assess recovery. Clinical and radiological evaluations were performed 2 years after surgery to detect incisional hernias. Quality of life was assessed using the RAND-36, Activities Assessment Scale (AAS), and PROMIS questionnaires at both follow-up points.

### Participants

Inclusion criteria was midline emergency laparotomy for any abdominal indication. Conversion from laparoscopy to laparotomy was accepted provided there was a written consent received prior the operation. Exclusion criteria were as follows:

- Previous ventral hernia repair with mesh in the midline
- World Health Organization (WHO) class of physical activity 3–4 (rest time greater than 50 per cent of day in bed) [11].
- Relaparotomy within 30 days of previous abdominal surgery
- Indication for laparotomy is hernia-related
- Pregnant or suspected pregnancy
- Patient is <18 years old
- Metastatic malignancy of any origin
- Patients living geographically distant and/or unwilling to return for follow-ups
- No informed consent provided
- Patient participates in other RCT (non-gastrointestinal trials were accepted)
- Planned or existing ostomy

Additionally, there were intra-operative exclusion criteria applied for both randomization groups as follows:

- Abdomen was left open
- Second-look laparotomy was planned
- Inability to keep the mesh securely out of the peritoneal cavity or close the anterior fascia
- Intra-abdominal non-curable malignancy diagnosed during the operation
- ≥2 cm hernia in midline

### Intervention

Onlay mesh placement has been associated with increased risk of seroma [9]. To avoid that complication in contaminated surgical site, we decided to use the retrorectus space for mesh, despite the likelihood of being more technically challenging and time consuming. A 8 cm-wide self-gripping polyester mesh (Progrip™, Medtronic, Sofradim Production, France) was chosen as the mesh has an indication for hernia prevention and does not need additional suturing.

In the control group, fascial closure was performed using the 4:1 small stitch technique with a continuous slowly absorbable monofilament suture in one layer.

In the mesh group, the posterior rectus sheath was opened extending the retrorectus opening both cranial and caudal to incision. The posterior layer was closed using continuous slowly absorbable monofilament suture with the 4:1 small stitch technique. An 8 cm-wide mesh was then applied over the closed posterior sheath, with gripping material directing posteriorly. The length of the mesh was cut to extend over the edges of incision. The anterior rectus sheath was closed using the slowly absorbable monofilament suture and 4:1 small stitch technique.

A step-by-step photographic guide of the surgical technique was provided to all participating centers to standardize the procedure.

### Outcomes

The primary outcome was the occurrence of incisional hernia (IH) detected either clinically or radiologically during the 2-year follow-up period.

The secondary outcomes included:

- Comprehensive Complication Index (CCI) within 30 days after surgery
- Surgical site infection (SSI) within 30 days of follow-up
- Fascial dehiscence within 30 days from surgery
- IH rate at 5 years
- IH repair rate within 2 and 5 years from surgery
- Reoperations due to mesh or hernia within 2 and 5 years
- Quality of life (QOL) assessed using the RAND-36, Activities Assessment Scale (AAS), and PROMIS questionnaires at 30 days, 2 years, and 5 years
- Health-economic exploratory measures, including
  - Time to create the retrorectus space and insert the mesh
  - Length of hospital stay
  - Material costs of abdominal closure
  - Duration of patient sick leave
  - Direct hospital costs due to recurrence or reoperation

Patients who were retired or stay-at-home caregivers were excluded from the sick leave assessment, as its duration could not be reliably estimated.

Adverse events and harms were systematically recorded and evaluated throughout hospitalization and during follow-up visits. All postoperative complications, including SSIs, seromas, hematomas, wound dehiscence, mesh-related complications, and deaths, were documented and graded according to the Clavien–Dindo classification, and summarized using CCI. The patients were clinically assessed during hospitalization by a surgeon who had not performed the operation, in order to maintain blinding.

A definition by the European Hernia Society for IH was applied [12]. A surgical site infection (SSI) was defined and documented according to the Centers for Disease Control and Prevention (CDC) criteria [13]. The RAND-36 is a validated quality-of-life instrument available in both official languages of Finland (Finnish and Swedish). The AAS and PROMIS questionnaires were selected to assess activity levels and functional outcomes, although they are not validated in the target languages. The results of all quality-of-life instruments were compared between randomized groups at 30-day and 2-year follow-up points.

### Blinding

Study participants were blinded to their randomized group throughout the entire follow-up period. The surgeon responsible for evaluating outcomes during hospital stay, and at the 30-day, 2-year, as well as the radiologist interpreting imaging, were also be blinded to group allocation. To preserve blinding, the medical records included only the statement, “Fascial closure was performed according to randomized group,” without disclosing which group the patient was assigned to.

The randomization number assigned to each patient was recorded in the medical files. Sealed envelopes labeled with the randomization numbers and containing the actual allocation group were accessible at all times, in case group information was required, for example, due to complications. If blinding was unintentionally broken, such event was documented.

### Sample Size Calculation and Statistical Analysis

The sample size calculation was based on an expected incisional hernia rate of 10% in the mesh group and 25% in the control group, as suggested by earlier studies [1–4]. Using a significance level (α) of 0.05 and a power of 80%, a minimum of 97 patients per group was required. With an anticipated dropout rate of 20%, the final target was 122 patients per group. The sample size calculation pertained only to the primary outcome, while the secondary outcomes were interpreted as hypothesis-generating.

Randomization was stratified to control for possible confounding factors. Stratification was based on body mass index (BMI less than 30 kg/m^2^ vs. 30 kg/m^2^ or higher), previous midline laparotomy (yes or no), conversion from laparoscopic to open surgery (yes or no), and age (below or above 65 years). Within each stratum, block randomization was performed using random permuted blocks of varying sizes (2, 4, 6, or 8). A separate randomization list was created for each participating center. Patients were randomly assigned in a 1:1 ratio to either the mesh or control group using a computer-generated list compiled by a biostatistician independent of clinical care.

The study data was stored in a secure electronic database that also included a built-in randomization software. The randomization result was visible only to the investigator who performed the randomization.

The primary outcome, defined as the incidence of incisional hernia within 2 years, was compared between groups using a 95% confidence interval for the difference. Categorical variables, including the primary endpoint, were analyzed using the chi-squared test or Fisher’s exact test. Continuous variables were compared using the Student’s t-test or Welch’s test, depending on whether the assumption of homogeneity of variance holds. The results for the above mentioned analyses were presented as difference between groups with 95% confidence interval (95% CI). Analyses were performed according to ITT principle.

Repeated measures data was analyzed using linear mixed model (LMM) using patients as random effect and time, group and time × group interaction as fixed effects. The results for the LMM were presented as difference between means with 95% CI.

As previous research on synthetic mesh utilized as prophylaxis at emergency midline laparotomy was scarce, an analysis of the complications and risks was planned for safety reasons after 30 patients would have been randomized to each group and reached 30 days’ follow-up.

Statistical analyses was conducted using IBM SPSS Statistics (Version 24.0) and SAS (Version 9.4).

## Results

**FIGURE 1:**
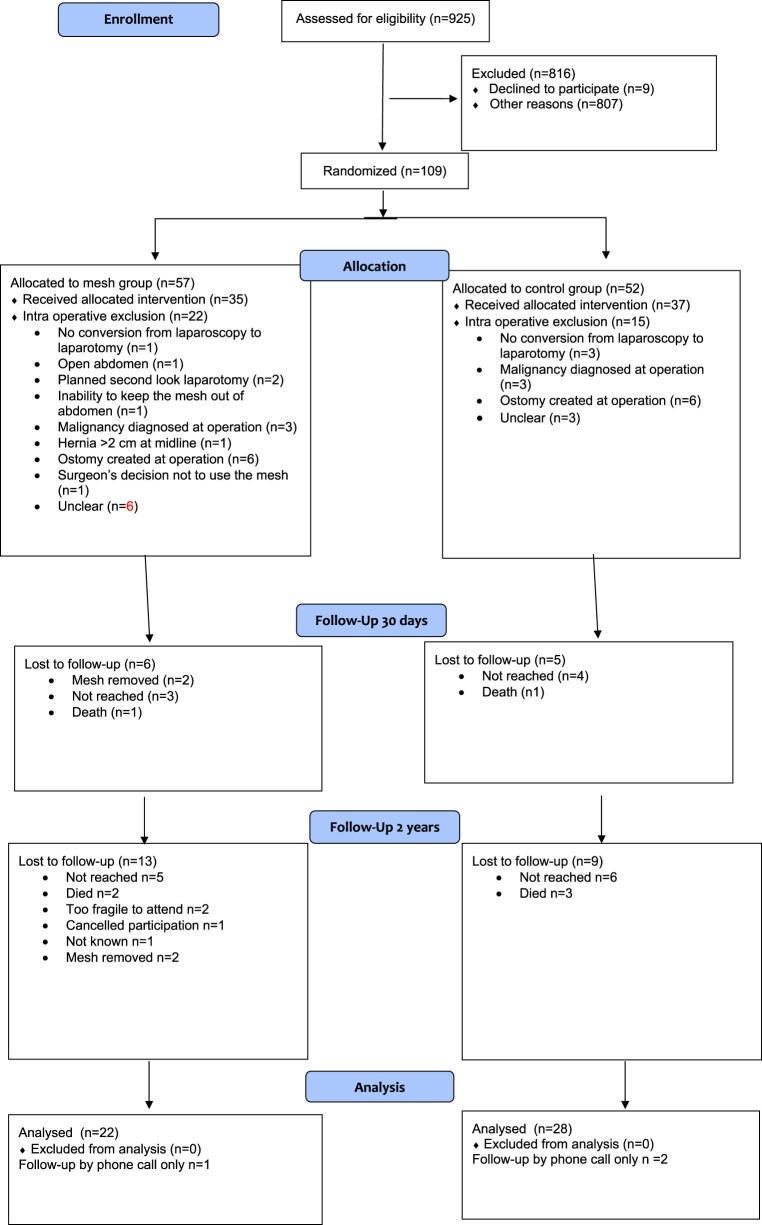
Flow chart.

The Preemer study faced problems in patient recruitment. The patient number was non achievable, and the enrollment was prematurely terminated. As the original patient number was evidently non achievable, the enrollment was prematurely terminated. Between April 2020 and October 2022, a total of 925 patients undergoing emergency midline laparotomy were screened at the participating hospitals. Out of that patient population, 109 patients were randomized (Figure 1). After randomization, 35 patients in the mesh group and 37 patients in the control group received the allocated intervention (Table 1). The details of surgical procedures, including contamination classifications, are presented in Table 2. One patient was re-operated for a seroma during primary stay, with mesh removal during the on-call hours (Table 3). There were no differences in the length of hospital stay or complications during the index stay. No fascial dehiscense were observed in either group. There was one (1/35, 3%) deep surgical site infection in mesh group compared to two (2/37, 6%) in control group (p = 0.513).

**TABLE 1:** Patient demographics.

	Mesh group (n = 35)	Control group (n = 37)
Sex		
Female	12 (34)	25 (68)
Male	23 (66)	12 (32)
BMI	27.0 ± 4.8	25.7 ± 4.9
Age	66.9 ± 14.7	68.7 ± 13.5
Smoking status		
Yes	3 (9)	3 (8)
No	28 (80)	29 (78)
Previously	3 (9)	3 (8)
ASA		
1	4 (11)	3 (8)
2	12 (34)	10 (27)
3	16 (46)	18 (49)
4	3 (9)	5 (14)
5	0	1 (3)
Laboratory results		
Creatine	84.7 ± 47.6	83.2 ± 37.6
Albumine	29.4 ± 6.6	32.2 ± 6.1
Cardiovascular disease	4 (11)	4 (11)
Congestive heart failure	1 (3)	3 (8)
COPD	7 (20)	5 (14)
ASO	6 (17)	7 (19)
Celebrovascular disease	3 (9)	2 (5)
DM	7 (20)	6 (16)
DM with end organ damage	2 (6)	3 (8)
Renal disease	3 (9)	2 (5)
Prior malignancy	2 (6)	2 (5)
WHO scale of activity		
1	22 (63)	26 (70)
2	12 (34)	7 (19)
3	1 (3)	4 (11)
Medications affecting healing		
Corticosteroid	5 (14)	4 (11)
Immunosupression	2 (6)	1 (3)
Biological medication	1 (3)	1 (3)
Previous incision		
Upper midline	1 (3)	3 (8)
Midline	1 (3)	4 (11)
Lower midline	3 (9)	8 (22)
Right subcostal	0	1 (3)
Bilateral subcostal	1 (3)	0
McBurney	4 (11)	1 (3)
Phannenstiel	2 (6)	3 (8)
Other	4 (11)	3 (8)
Previous hernia		
Umbilical	5 (14)	1 (3)
Inguinal	2 (6)	0

Nominal variables are reported as counts and percentages (in parentheses); continuous variables are reported as means and standard deviations. BMI, body mass index; ASA, american society of anesthesiologists physical status classification; COPD, chronic obstructive pulmonary disease; ASO, arteriosclerosis obliterans; DM, diabetes mellitus; WHO, world health organization.

**TABLE 2:** Operation details.

	Mesh group (n = 35)	Control group (n = 37)	P value	Difference (95% CI)
Operation time (min)	103.7 ± 36.5	96.1 ± 52.4	0.236	
Contamination class			0.578	
1 Clean	1 (3)	2 (5)		
2 Clean-Contaminated	22 (63)	25 (68)		
3 Contaminated	6 (17)	4 (11)		
4 Dirty/Infected	6 (17)	6 (16)		
Primary operation			0.200	
Small bowel resection	16 (46)	13 (35)		
Colon resection	6 (17)	9 (24)		
Division of adhesive band in intestinal obstruction	6 (17)	5 (14)		
Adhesiolysis	2 (6)	6 (16)		
Explorative laparotomy	3 (9)	0		
Cholecystectomy	0	1 (3)		
Gastric or duodenal ulcer suturing	1 (3)	2 (5)		
Small bowel suturing	0	1 (3)		
Vaginal suturing	1 (3)	0		
Length of the midline incision (cm)	16.1 ± 4.6	16.9 ± 4.5	0.243	
Length of suture material used (cm)	77.1 ± 27.0	75.0 ± 28.6	0.371	2.1 (−15.6 to 11.1)
Suture material to wound length ratio	4.9 ± 1.7	4.5 ± 1.5	0.130	
Blood loss (cc)	100.2 ± 109.9	81.6 ± 102.1	0.246	
Time to create the retrorectus space and apply the mesh (min)	20.9 ± 10.2	n/a		
The average cost of mesh per patient (€)	235	n/a		
Length of stay (days)	6.5 ± 3.6	6.1 ± 2.3	0.390	0.4 (−2.8 to 1.1)

Nominal variables are reported as counts and percentages (in parentheses); continuous variables are reported as means and standard deviations.

CI, confidence interval; IH, incisional hernia, cm cm, cc cubic centimeters.

**TABLE 3:** 30 days′ follow-up.

	Mesh group (n = 29)	Control group (n = 32)	P value	Difference (95% CI)
Type of follow-up			0.548	
Visit	10 (38)	9 (28)		
Call	19 (66)	23 (72)		
Complications				
Fascial dehiscense	0	0	n/a	
Superficial SSI (C-D 3b)	1 (3)	0	0.491	3.6 (−8.5–17.7)
Deep SSI	1 (3)	2 (6)	0.513	−3.3 (−18.7 to 11.7)
Clavien-dindo Classification 2	1 (3)	1 (3)	0.667	0.1 (−13.9–14.5)
Clavien-dindo Classification 3a	0	1 (3)	0.491	−3.4 (−17.2 to 8.9)
Mesh related complications	0	0		
Mesh removed during hospital stay	1 (3)	n/a		
Place of further care			0.232	
Home	23 (82)	24 (83)		
Healthcare center	2 (7)	5 (17)		
Other hospital	1 (3)	0		
Other	2 (7)	0		
Returned to previous level of activity	25 (76)	26 (81)	0.432	−0.4 (−18.2 to 17.1)
Returned to work	12 (41)	12 (38)	0.562	1.5 (−22.8–25.5)
Length of sick leave	36.6 ± 9.7	45.0 ± 18.5	0.268	−8.4 (−30.4–46.9)
Wound status			0.366	
Healed	28 (97)	31 (97)		
Open less than 2 cm	0	1 (3)		
Open more than 2 cm	1 (3)	0		
Readmission to hospital	3	2 (6)	0.468	3.8 (−12.8–21.0)
Hematoma, COPD worsening	1 (3)	0		3.6 (−8.5–17.7)
Fever	2 (7)	0		7.1 (−5.6–22.6)
Pulmonary embolism	0	1 (3)		−3.4 (−17.2 to 8.9)
Deep SSI	0	1 (3)		−3.4 (−17.2 to 8.9)
CCI	23,9 ± 4,8	22.0 ± 2.2	0.151	1.9 (−5.7 to 1.9)

Nominal variables are reported as counts and percentages (in parentheses); continuous variables are reported as means and standard deviations.

SSI, surgical site infection, C-D Clavien-Dindo Classification, COPD, Chronic Obstructive Pulmonary Disease; CCI, comprehensive complication index.

At the 30-day follow-up, 29 patients in the mesh group and 32 patients in the control group were successfully contacted and interviewed regarding their recovery by phone call or visit to outpatient clinic (Table 3). During the 30 days’ follow-up, one patient (1/29, 3%) in the mesh group had been readmitted due to retrorectus hematoma that was later drained (Clavien-Dindo Complications class 3a). There was no difference in the Comprehensive Complications Index between the groups. There were two deaths, one in both groups. To our knowledge, the deaths were not related to mesh. Notably, some patients who could not be reached at the 30-day follow-up were later reached at the 2-year follow-up.

By the 2-year follow-up point, 22 patients in the mesh group and 28 in the control group were clinically evaluated (Table 4). In the control group, 1/22 (4%) patient had a clinically detectable incisional hernia, which was also visible on ultrasound, although the hernia was asymptomatic. A patient (5%) in the mesh-group who did not attend the clinical follow-up but had been diagnosed with an abdominal wall infection with an enterocutaneous fistula and had a mesh removal (Clavien-Dindo classification 3b). Additionally, one (5%) patient experienced an internal hernia, requiring emergency laparotomy when a loop of intestine herniated between the mesh and the peritoneum (Clavien-Dindo Classification 3b).

**TABLE 4:** 2 years’ follow-up.

	Mesh group (n = 22)	Control group (n = 28)	P value	Difference (95% CI)
Sex			0.053	26.9 (−0.6–49.6)
Female	9 (41)	19 (68)		
Male	13 (55)	9 (32)		
Age	66.4 ± 15.0	70.4 ± 14.3	0.168	
Follow-up time (months)	26.3 ± 3.6	26.3 ± 2.9	0.460	
Operation since index surgery	1 (5)	1 (4)	0.691	1.0 (−13.7–18.5)
IH at clinical evaluation	0	1 (4)	0.560	−3.6 (−17.7 to 11.6)
IH at ultrasound	0	1 (4)	0.560	−3.6 (−17.7 to 11.6)
Blinding of the patient maintained			0.493	
Yes	20 (91)	26 (93)		−1.9 (−21.4 to 14.9)
No	1 (5)	0		
Not known	1 (5)	2 (7)		
Blinding of the surgeon evaluating the patient maintained			0.439	
Yes	18 (82)	21 (75)		6.8 (−17.0–28.2)
No	4 (18)	5 (18)		
Not known	0	2 (7)		

Nominal variables are reported as counts and percentages (in parentheses); continuous variables are reported as means and standard deviations.

The time required to open the retrorectus space, close the posterior layer and apply the mesh was 20.9 ± 10.2 (range 8–53 min, SD 10.0). The average cost for the mesh was 235€. Additionally, there were in total 3 complications in mesh group requiring further procedures. The length of sick leave was similar between the groups. There was no difference in quality of life between the groups (Supplementary Material).

## Discussion

This randomized controlled trial found no difference in the rate of incisional hernia between patients who received retrorectus mesh and those whose fascia was closed with a standard 4:1 small-stitch technique. These results are in contrast with earlier studies involving both emergency and elective midline laparotomies, in which prophylactic mesh has significantly reduced incisional hernia rates [2, 5–8]. However, the IH rate was significantly lower in control group closed with 4:1 small stitch closure in this study, compared to significantly higher IH rates previously reported [1–8].

During the 2-year follow-up, two patients experienced complications directly attributable to the creation of the retrorectus space. These types of mesh related complications have not been commonly reported in earlier trials.

The increased costs in the mesh group were the time required to apply the mesh and the cost of the mesh. The mesh cost 235€ on average per patient. Additionally, there were additional procedures required in the mesh group causing more costs. As the rate of IH was very low, the mesh use did not lead to savings in healthcare.

The trial was prematurely terminated due to significant challenges in patient recruitment. This reflects not only difficulties to recruit the emergency patients and the still existing hesitations to use the mesh in contaminated surgical site despite the evidence, but also the shift from emergency midline laparotomies to laparoscopies. Consequently, the small sample size limits the generalizability of the findings and precludes firm conclusions. Furthermore, a large proportion of patients were excluded intraoperatively or lost to follow-up, underscoring the difficulties of conducting high quality randomized controlled trials in emergency surgical settings.

The results of this trial leave more questions than give answers. Further trials are needed to comparing small stitch technique to mesh prevention, possibly concentrating on patients with increased risk of IH in addition to emergency laparotomy alone. The retrorectus placement of the mesh may carry an increased risk for complications.

## Data Availability

The datasets generated and/or analyzed during the current study are not publicly available due to Finnish laws on privacy protection but are available from the corresponding author on reasonable request. Requests to access the datasets should be directed to Elisa EM, elisa.makarainen@pohde.fi.
