# Supplementary material for: Prophylactic Retrorectus Mesh Versus Small-Stitch Closure After Emergency Midline Laparotomy: 2-Year Results of a Randomized Controlled Trial
Source: J Abdom Wall Surg. 2025 Nov 27;4:15500. doi: 10.3389/jaws.2025.15500 (PMC12695720; doi:10.3389/jaws.2025.15500)
Supplement: Supplementary file 1 [file Supplementaryfile1.docx]

| **Supplementary Material**  **Quality of life RAND-36** |  |  |  |  |  |  |
| --- | --- | --- | --- | --- | --- | --- |
| Mean scores |  | Mesh group | Control group | P group | Difference  (95% confidence  interval) | p group |
| Mental health |  |  |  |  |  |  |
|  | Discharge | 68.5 (20.9) | 64.3 (20.9) |  |  |  |
|  | 30 days | 77.3 (15.6) | 64.3 (15.8) | 0.035 | 13.0 (0.9 to 24.5) | 0.59 |
|  | 2 years | 78.5 (18.2) | 81.2 (13.8) | 0.921 | -25.5 (-13.1 to 11.9) | 0.83 |
| Role emotional |  |  |  |  |  |  |
|  | Discharge | 56.2 (45.4) | 63.9 (40.4) |  |  |  |
|  | 30 days | 55.6 (47.1) | 47.9 (48.6) | 0.932 | 13.0 (-22.1 to 34.4) | 0.74 |
|  | 2 years | 66.7 (45.1) | 92.2 (18.7) | 0.452 | -25.5 (-62.1 to 1.8) | 0.54 |
| Social functioning |  |  |  |  |  |  |
|  | Discharge | 56.5 (24.4) | 72.5 (25.8) |  |  |  |
|  | 30 days | 65.8 (31.4) | 63.3 (25.2) | 0.481 | -11.3 (-11.3 to 23.7) | 0.32 |
|  | 2 years | 79.8 (25.6) | 81.6 (26.6) | 0.803 | -5.4 (-21.1 to 16.4) | 0.19 |
| Vitality |  |  |  |  |  |  |
|  | Discharge | 56.1 (22.1) | 49.6 (22.0) |  |  |  |
|  | 30 days | 61.7 (23.3) | 50.6 (16.2) | 0.088 | 11.1 ( -1.8 to 26.4) | 0.32 |
|  | 2 years | 66.9 (21.8) | 68.2 (19.7) | 0.781 | -1.3 (-17.2 to 13.0) | 0.19 |
| General health |  |  |  |  |  |  |
|  | Discharge | 62.7 (22.2) | 53.2 (19.5) |  |  |  |
|  | 30 days | 54.2 (24.4) | 57.3 (16.3) | 0.769 | -3.1 (-10.5 to 14.2) | 0.19 |
|  | 2 years | 62.9 (16.9) | 62.8 (15.9) | 0.457 | 0.1 (-17.7 to 8.0) | 0.80 |
| Bodily pain |  |  |  |  |  |  |
|  | Discharge | 32.7 (30.1) | 39.7 (26.6) |  |  |  |
|  | 30 days | 57.7 (22.7) | 53.9 (26.3) | 0.609 | 0.6 (-12.5 to 21.2) | 0.32 |
|  | 2 years | 81.2 (18.4) | 72.9 (18.9) | 0.396 | 1.6 (-10.3 to 25.8) | 0.38 |
| Physical function |  |  |  |  |  |  |
|  | Discharge | 37.7 (30.7) | 52.5 (32.2) |  |  |  |
|  | 30 days | 55.0 (33.4) | 58.4 (25.1) | 0.959 | -3.4 (-4.3 to 13.6) | 0.030 |
|  | 2 years | 82.4 (22.8) | 85.0 (19.6) | 0.711 | -2.6 (-23.7 to 16.2) | 0.24 |
| Role physical |  |  |  |  |  |  |
|  | Discharge | 41.3 (46.2) | 33.0 (42.5) |  |  |  |
|  | 30 days | 29.2 (42.2) | 17.2 (31.3) | 0.244 | 6.5 (-11.1 to 43.3) | 0.78 |
|  | 2 years | 77.6 (35.1) | 82.4 (36.2) | 0.496 | -3.2 (-39.2 to 19.1) | 0.23 |
|  |  |  |  |  |  |  |
| Preoperative and postoperative mean scores for each study group are shown for the eight  dimensions assessed by the RAND-36 health-related quality of life questionnaire.  Values are mean (standard deviation).  P- value and the between group difference with confidence interval are calculated by linear mixed model. | | | | | | |

| **Supplementary Material**  **Quality of life**  **Activities Assessment Scale** |  | Mesh group | Control group | P group | Difference  (95% confidence  interval) |
| --- | --- | --- | --- | --- | --- |
|  | Discharge | 72.9 (19.6) | 74.5 (16.9) |  |  |
|  | 30 days | 76.8 (17.9) | 81.7 (13.3) | 0.244 | -4.9 (-14.5 to 5.8) |
|  | 2 years | 90.8 (9.1) | 85.0 (13.6) | 0.496 | 8.8 (-5.6 to 16.2) |

Preoperative and postoperative mean scores for each study group are shown for Activities Assessment Scale health-related quality of life questionnaire.

Values are mean (standard deviation). P- value and the between group difference with confidence interval are calculated by linear mixed model.
